# Bio-Guided Extraction of a Phenolic-Rich Extract from Industrial Peanut Skin with Antioxidant and Hypotensive Potential

**DOI:** 10.3390/foods13213410

**Published:** 2024-10-26

**Authors:** Ana Carla de Matos, Daniel Batista, Luiza Gabriella Soares Dantas Pinheiro, Gabriela de Matuoka e Chiocchetti, Paulo Roberto de Araújo Berni, Gabriela Alves Macedo, Juliana Alves Macedo

**Affiliations:** 1 Department of Food and Nutrition, Faculty of Food Engineering, Universidade Estadual de Campinas (UNICAMP), Campinas 13083-862, Brazil; anac.dematos@gmail.com (A.C.d.M.); d070524@dac.unicamp.br (D.B.); luizagdantas15@gmail.com (L.G.S.D.P.); macedoga@gmail.com (G.A.M.); jumacedo@gmail.com (J.A.M.); 2 Department of Food Science, Federal University of the State of Rio de Janeiro (UNIRIO), Rio de Janeiro 22290-250, Brazil

**Keywords:** phenolic compounds, peanut skin, up-cycling, antioxidant, ACE inhibition

## Abstract

Peanut composition includes phenolic compounds, especially in the skins, which are often not consumed. High blood pressure affects more than one billion people worldwide and is considered a high-risk factor for cardiovascular diseases. Several studies have correlated antihypertensive activity with the total phenolic content present in the plants. This study evaluated the hydroethanolic extraction of phenolic compounds from the industrial residue of peanut skin and evaluated the antioxidant and antihypertensive capacity of these extracts using in vitro models. A rotational central composite design (DCCR) was proposed to study the influence of the variables: (1) the ethanol concentration on the hydroalcoholic extractor solution, and (2) the proportion of solid sample (waste) per liquid in the extraction (mass/volume) in a simple solid—a liquid extraction process. The optimal extraction conditions within this model were 50% ethanol in water, and the proportion of sample to extraction solution (*m*/*v*) equaled to 0.2. The extract obtained had significant antioxidant capacity, both in chemical (ORAC) and in cellular models, with potential for free radical scavenging. Significant levels of ACE inhibition were also found, indicating antihypertensive activity.

## 1. Introduction

The peanut (*Arachis hipogaea* L.) production industry handles millions of tons of products annually worldwide. Historically, its production has mainly focused on the nut and oil extraction, resulting in tons of waste throughout the production chain. The kernels are used to make peanut butter, peanut confections, and peanut oil, and it is estimated that 35 to 45 g of peanut skin is produced per kg of shelled peanut kernel [1]. These peanut skins are usually discarded or used in animal feed, although they are rich in bioactive compounds such as polyphenols and phenolic acids.

Polyphenols are a group of secondary metabolites found in plants and distinguished for their antioxidant activity. They act on redox imbalance, helping to combat damage caused by excess free radicals, which are the etiology of all chronic non-communicable diseases. The growing awareness of the importance of bioactive compounds in the diet has also sparked interest in identifying alternative and sustainable sources of these compounds, aiming to minimize food waste and promote a circular economy. In this context, agricultural by-products, such as peels, seeds, and leaves, which would typically be discarded, have been explored as potential sources of bioactive compounds [2,3]. The health benefits of polyphenols include the protection of cells against oxidative damage by free radical reduction [4], and decreasing blood pressure, improving blood vessel function [5].

Hypertension is the occurrence of systolic blood pressure equal to or more than 140 mmHg and diastolic blood pressure equal to or more than 90 mmHg (140/90 mmHg) and affects more than 1.2 billion people in the world, according to World Health Organization database [6]. An increased effort in blood distribution by the heart is considered a risk factor for cardiovascular disease occurrence, making hypertension one of the biggest public health problems, with more than 9 million deaths per year estimated as being caused by hypertension complications in the latest published report [7]. Among the mechanisms for blood pressure (BP) control, the Renin–Angiotensin–Aldosterone System (RAAS) is one of the most important. This mechanism acts through vascular remodeling and sodium and water reabsorption, leading to BP elevation. As a result, RAAS is the most popular target for hypertension treatment, mainly by angiotensin-I-converting enzyme inhibition, which breaks the RAAS cascade reaction [8,9]. In popular medicine, it is possible to find recommendations of plants, fruits, and herbs for blood pressure control [10]. Many studies correlated the antihypertensive potential of those plants to their phenolic content and the ability of these compounds to modulate RAAS, mainly by ACE inhibition [11,12,13,14,15].

Based on the above, the objectives of this work were to enable an environmentally friendly extraction process, bioguided by the amount and antioxidant activity of the phenolic compounds in the peanut skin residue. In addition, the in vitro hypotensive activity of the extract was also evaluated.

## 2. Materials and Methods

### 2.1. Sample Acquisition

Peanut skins (Valencia variety) were obtained from the food processing industry located in Tupã (São Paulo, Brazil), in December 2018. Samples were ground with a portable laboratory grinder (Blender OBL10/2, Machesoni, Sao Paulo, Brazil), settled in a foil laminated sealed pack, protected from light to avoid a possible degradation of the phenolic compounds, and stored under −80 °C until extraction. The proximate composition of peanut skin was determined in triplicate following the AOAC method [16]. Briefly, the moisture content was measured by evaporating water from the sample in an oven at 105 °C for 14 h. To determine the ash content, the sample was calcined in a muffle furnace at 550 °C for 4 h, eliminating organic matter. Protein was analyzed using the micro-Kjeldahl method, which involves acid digestion and distillation. Lipids were extracted with the Soxhlet method using ethyl ether and were quantified by the difference in mass after evaporation of the solvent. Fibers were determined using the enzymatic–gravimetric method, and the carbohydrate content was calculated using the difference.

### 2.2. Study to Improve the Extraction of Phenolic Compounds—Central Rotational Composite Design (DCCR)

To determine the best conditions for extracting phenolic compounds from peanut skin residue, a conventional solid–liquid extraction process was carried out, with an optimization study of the process variables through a central rotational composite design (DCCR). The two independent variables were defined as the ethanol concentration of the hydro-alcoholic extraction solution (% *v*/*v*) and the proportion of solid sample to liquid in the extraction (*m*/*v*); the dependent variables considered were total phenolic compound (TPC) content (µmol gallic acid equivalent/mg dry extract) and ORAC value (μmol equivalent of Trolox/g dry extract). The independent variables were coded according to Equation (1).

(1) $$xi=\frac{\left(Xi-X0\right)}{\Delta Xi}$$

in which *xi* is the coded variable referring to an evaluated process parameter, *Xi* is the natural variable of the process parameter, *X*0 is the natural value of the variable at the central point, and ∆*Xi* is the variation in the value between the points. The natural variables and those coded at each level are presented in Table 1.

The Protimiza Experimental Design software (online version, Campinas, São Paulo, Brazil) (https://experimental-design.protimiza.com.br/, accessed on 30 March 2022) defined the DCCD with 2 factors as the combination of 8 trials, plus the replicates of the central point (06 cubic points, 02 star points, and 05 replicates at the center point to estimate the experimental error and to investigate the suitability of the proposed model).

In practice, the tests were carried out by preparing mixtures of powdered peanut skin waste and hydroalcoholic solution under different conditions, and these were mechanically shaken at 200 rpm for 2 h at 25 °C. After this step, the extracts were filtered and concentrated in a rotary evaporator to remove ethanol (at 40 °C for 2 h 30 min). The extracts were then frozen, freeze-dried, and stored at −20 °C until the dependent variables (TPC and ORAC) were analyzed as described next.

### 2.3. Extract Characterization

#### 2.3.1. Total Phenolic Compounds (TPCs)

TCP was determined using the Folin–Ciocalteu method [17]. A standard curve of gallic acid was constructed (16–300 μg/mL). All analyses were performed in triplicate, and the results are expressed as milligrams of gallic acid equivalent (GAE) per milligram of dry extract.

#### 2.3.2. UPLC-ESI-QTOF-MS/MS Analysis

UPLC-ESI-QTOF-MS/MS analysis was carried out on a Waters modular UHPLC system, consisting of an Acquity^®^ HClass QSM quaternary pump, an Acquity^®^ Sample Manager—FTM autosampler, and an Acquity^®^ PDAeλ diode array detector, coupled to a Waters Xevo^®^ G2-XS mass spectrometer equipped with an electrospray ionization (ESI) source, a quadrupole analyzer, and a collision cell in sequence with a time-of-flight (TOF) analyzer. Samples were dissolved in an 80% (*v*/*v*) methanol/water solution. A 0.1% (*v*/*v*) formic acid solution in ultrapure water was used as mobile phase component A, and acetonitrile acidified with formic acid at 0.1% (*v*/*v*) as component B. Chromatographic runs were performed at a flow rate of 0.350 mL/min, starting with 5% of component B in the mobile phase composition. The elution gradient was programmed to increase the %B until reaching 100% within 10 min. The %B was kept constant at 100% for 4 min, then returned to 5% within 1 min, remaining constant for 5 min to allow column reconditioning. The analyses were performed on a Waters Acquity^®^ UPLC HSS T3 reversed-phase (C18) column with 1.8 μm particles, 2.1 mm internal diameter, and 100 mm length, maintained at a temperature of 40 °C throughout the process.

Full scan mass spectra and MS/MS were acquired in negative mode. The electrospray capillary and transfer cone voltages were maintained at 2.5 kV and 20 V, respectively. The ESI source and desolvation gas temperatures were set at 120.0 °C and 350.0 °C, respectively. MS/MS spectra were acquired in Fast DDA (data-dependent acquisition) mode, where only ions with intensities above 100 were fragmented. Ion fragmentation was performed using a voltage ramp, where for low m/z values, the energy ranged from 10 to 40 eV, and for high *m*/*z* values, it ranged from 50 to 80 eV. The acquisition range was from *m*/*z* 100 to 1500.

#### 2.3.3. Antioxidant Potential in Vitro

The ORAC values were determined according to Dávalos et al. [18]. The sample was diluted in phosphate buffer 75 mM, pH 7.4. Trolox^®^ (6-hydroxy-2,5,7,8-tetramethylchrome-2-carboxylic acid, AcrosOrganics, Geel, Belgium) was prepared as a reference standard at concentrations between 1.5 and 1500 μM in 75 mM phosphate buffer, pH 7.4. Then, 20 µL aliquots of the sample, Trolox solution, or buffer (blank) were distributed in a 96-well plate (black and opaque) followed by the addition of 120 µL of fluorescein sodium salt solution 0.38 μg/mL (Ecibra, São Paulo, Brazil) diluted in phosphate buffer 75 mM, pH 7.4. The reaction was started by adding 60 µL of 2,2′-Azobis(2-methylpropionamidine) dihydrochloride (AAPH) radical solution (Sigma-Aldrich, Steinheim, Germany) at a concentration of 108 mg/mL dissolved in phosphate buffer 75 mM, pH 7.4. Fluorescence was monitored every 56 s for 75 min, in a Fluostar Optimo Fluorimeter (BMG LABTECH, Ortenberg, Germany) at 37 °C and with a 485nm excitation and 520nm emission filter. The analysis was performed in triplicate, and the results were expressed in μM equivalent of Trolox (ET)/g dry extract.

The DPPH (2,2-diphenyl-1-picrylhydrazyl) analysis was performed according to Peschel et al. [19] with modifications by Macedo et al. [20]. Three different concentrations of samples were prepared in 70% methanol. Trolox was prepared as a reference standard at concentrations between 15 and 300 μM/L in 70% methanol, and the reference blank was prepared with ultra-pure water. The reaction medium consisted of 50 µL of sample/standard/blank arranged in transparent 96-well plates and 150 µL of DPPH solution (0.2 mM/L in 70% methanol). Absorbance was read in a Fluostar Optima Fluorimeter (BMG Labtech, Germany) with a 520nm absorption filter for 112 min. Results are expressed in μmol ET/g dry extract.

The FRAP (Ferric Reducing Antioxidant Power) assay was performed following the method of Benzie and Strain [21], with modifications. The FRAP reagent solution was prepared by mixing 2.5 mL of 0.3 M acetate buffer (pH 3.6), 250 μL of 10 mM tripyridyltriazine (TPTZ) in a 40 mM HCl solution, and 250 μL of a 20 mM ferric chloride solution. A 36 μL aliquot of the extract solutions was incubated with 270 μL of the FRAP reagent in a 96-well plate. For the calibration curve, Trolox solution (1.5 to 100 μg/mL) was used. Absorbance was measured at 595 nm for 10 min (7 cycles of 88 s) using a fluorimeter (FLUOstar Optima, BMG Labtech, Germany). The results were expressed as μmol ET/g dry extract.

#### 2.3.4. In Vitro ACE Inhibitory Activity

This method relies on the ability of ACE to hydrolyze a synthetic fluorogenic peptide, which releases a fluorescent compound, as demonstrated by Batista et al. [22]. A 25 μL solution of the extract (10 μg GAE/mL) was incubated with 25 μL of the kit enzyme solution (CS0002B) in a 96-well black plate for 5 min at 37 °C. The reaction was initiated by adding 50 μL of the substrate solution (CS0002C), and the fluorescence change was monitored using a fluorimeter (FLUOstar Optima, BMG Labtech, Germany) at 37 °C, recording measurements every minute for 5 min with an excitation filter set to 340 nm and an emission filter set to 405 nm. For determining the positive control activity of ACE, the extracts were substituted with assay buffer. A standard curve was created using the kit standard solution (CS0002D). All reagents were prepared in the assay buffer (CS0002A) at the recommended dilutions immediately before conducting the assay. The obtained data (in duplicate) were analyzed using the Excel-based calculation sheet provided by the manufacturer to determine ACE activity. The results were expressed as the percentage of ACE inhibition and calculated according to Equation (2).

(2) $$\%ACEi=\left(\frac{PC-Sp}{PC}\right)\times 100$$

where %*ACEi* represents the percentage of ACE inhibition, *PC* refers to ACE activity in the positive control (without inhibitors), and *Sp* denotes ACE activity with the samples (peanut skin extract). One unit of ACE is defined as the amount of enzyme required to release 1 nmol of the fluorescent compound from the substrate per minute under the assay conditions.

### 2.4. Intracellular Antioxidant Assay

#### 2.4.1. Caco-2 Cell Maintenance

Human intestinal epithelial cells, Caco-2, were cultured in 75 cm^2^ flasks in Dulbecco’s Modified Eagle Medium (DMEM, GiBCO Life Technologies, Waltham, MA, USA) supplemented with 1% antibiotics (penicillin/streptomycin, GiBCO Life Technologies) and 10% Fetal Bovine Serum (FBS, GiBCO Life Technologies). The cells were maintained at 37 °C with 5% CO_2_ and constant humidity. The culture medium was changed 2–3 times weekly, and cells were passaged at 75–85% confluence. Cell passages between 28 and 40 were used in the experiments.

#### 2.4.2. Cytotoxicity

The viability of Caco-2 cells was assessed through an MTT (3-[4, 5-dimethylthiazol-2-yl]-2,5-diphenyl-tetrazolium bromide) assay. Caco-2 cells (2 × 10^5^ cells/well) were seeded in 96-well plates. After 24 h, the culture medium was removed, and the cells were treated with peanut skin extracts in DMEM (without FBS) at the different concentrations (5–50 μg/mL) of the extracts. After 24 h, the culture medium was replaced with an MTT solution (0.5 mg/mL in PBS), and the cells were incubated at 37 °C for 2 h. Subsequently, the formed crystals were dissolved by adding 100 μL of DMSO, and the absorbance was measured at 570 nm. The results obtained are expressed as a percentage relative to the control.

#### 2.4.3. Generation of Intracellular Reactive Oxygen Species (ROS)

To measure the intracellular ROS content, a fluorescent probe sensitive to oxidants, CM-H_2_DCFDA(5-(and-6)-chlor omethyl-20,70-dichlorodihydrofluorescein diacetate) was utilized [23]. Caco-2 cells (3 × 10^4^ cells/well) were seeded in 96-well black wall microplates. After 24 h, the culture medium was replaced with Hanks’ Balanced Salt solution (HBSS, GiBCO Life Technologies) containing 10 μM CM-H_2_DCFDA and incubated for 30 min at 37 °C. After washing with PBS to remove any unabsorbed probe, the cells were treated with AAPH at a concentration of 50 μM (as a ROS generator) and peanut skin extracts in HBSS at 5 and 10 μg/mL concentrations for 5 h. AAPH was used as a positive control and an additional control group was made, without any treatment. Fluorescence readings were recorded at an excitation wavelength of 490 nm and an emission wavelength of 520 nm every 30 min. The results are presented as the percentage increase in fluorescence compared to the respective baseline value.

### 2.5. Statistical Analysis

For DCCR, the experimental results were fitted to a second-order polynomial function once the experiments had been performed. A Student *t*-test was used to determine the statistical significance of the regression coefficients, and the analysis of variances (ANOVA) was performed on the experimental data to evaluate the statistical significance of the model. The model for the response was expressed in terms of coded variables, without taking into account the statistically non-significant terms. The analysis was carried out using Protimiza Experimental Design online software (Campinas, São Paulo, Brazil) (https://experimental-design.protimiza.com.br/, accessed on 30 March 2022).

Results are expressed as mean ± standard deviation (SD). To evaluate the generation of ROS in Caco-2 cells, a one-factor ANOVA was performed, followed by a Tukey test, to compare the samples (*p* < 0.05). The experiments were carried out in at least triplicates, and the results were confirmed in a second independent experiment. All analyses and graphs were performed using the GraphPad Prism 8.0 software.

## 3. Results and Discussion

### 3.1. Proximate Composition of Peanut Skin

The proximate composition of the peanut skins was evaluated, and the results are shown in Table 2. These data are in accordance with the literature, which reports that peanut processing byproducts are a potential source of natural proteins, fibers, and lipids, as well as phenolic compounds [1].

Our results are similar to those found by Manrich et al. [24], the exception being the estimated lipid value (8.2 ± 0.8%), which was lower than the present study. Camargo et al. [25] found only 4.66 ± 0.04 of protein in peanut skin and 11.2 ± 0.31 of lipids. This variation may be related to the variety of peanut used and the processes that the sample underwent prior to analysis.

### 3.2. Bioguided Production of Bioactive Extracts

Some studies have evaluated the extraction of phenolic compounds from peanut skin in recent years using various solvents such as methanol, acetone, and ethyl acetate [26]. The present study used ethanol/water as the solvent as it is known to be efficient in extracting compounds of interest and is considered less toxic to humans and the environment. Furthermore, the optimization of multiple extraction parameters focused not on the extraction yield, but on the phenolic content and its antioxidant potential, representing a bio-guided approach to obtaining the most desirable extract. The real and coded levels of the two studied parameters are presented in Table 3, and these parameters are the percentage of ethanol in the water extraction solution, and the ratio of solid residue and extraction solution applied to the extraction process.

The ANOVA reproduced in Table 4 and Table 5 for each response variable—TPC and ORAC—showed that the models were significant. For the TF response, the Fisher F-test (F5;5 = 21.75 > Ft 0.9; 5;5 = 3.45) was 5–6 times higher than the Ft, demonstrating that this regression was statistically significant at a 95% confidence level. In addition, the R2 (multiple correlation coefficient) of the regression equation obtained was 0.95 (a value > 0.75 indicates the aptness of the model), which means that the model can explain 95% of the variation in the response. As for the ORAC response, the ANOVA presented an F-test (F5;5 = 9.13 > Ft 0.9; 5;5 = 3.45) that was also significant, demonstrating that this regression was also statistically significant at a 90% confidence level. The R2 obtained was 0.9.

**Figure 1:**
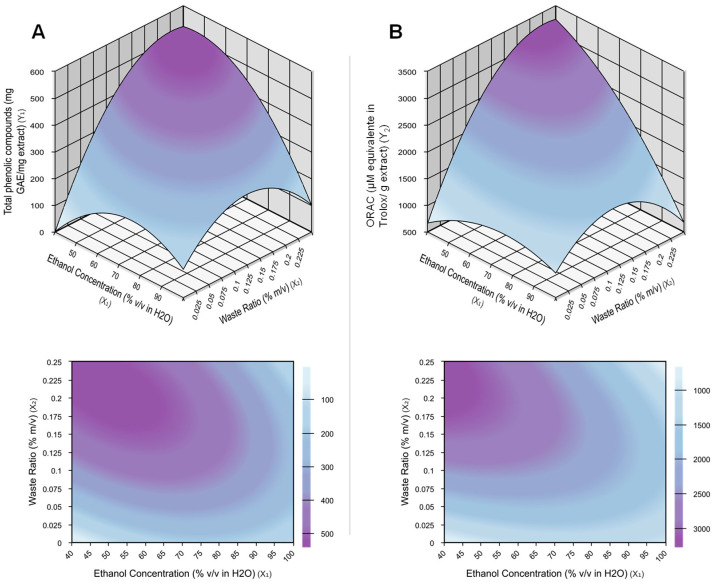
Contour curve and response surface for (**A**) total phenolics and (**B**) antioxidant activity—ORAC as a function of solvent and sample proportion in extraction, according to DCCR.

These significant models generated the response surfaces illustrated in Figure 1. Through the response surfaces generated, it is possible to observe that when reducing the percentage of ethanol in water and increasing the proportion of residue per solvent, the TPC and antioxidant activity tend to increase. The profile of trends concerning the two responses evaluated was quite similar, showing that there is a strong correlation between the response variables FT and ORAC. This correlation is as expected as antioxidant activity is directly proportional to the TPC extracted from the sample. Convergence of the increasing trends of the two response variables is useful for choosing the best extraction conditions in this study.

From the trends indicated by the model, the logic would be to expand the variation ranges of residue/solvent contents to values greater than the upper limit studied, and simultaneously, to reduce the concentration of ethanol in water to levels lower than those tested. However, in practice, there were limits to the increase in residue/solvent levels that needed to be respected. With ratios greater than 0.2, this material already had characteristics of very little free water, preventing the agitation process during extraction, and making subsequent filtration unfeasible. Given these limitations of the process conditions, we defined that the ratio of 0.2 was the limit, and therefore, the best value for this process parameter.

After reducing the ethanol concentration in water to below 50%, mono-variable tests at 40% ethanol revealed no significant difference in TPC compared to the 50% ethanol solution. However, the time required for rotary evaporation to remove ethanol in the next stage increased significantly. Thus, the optimal condition for phenolic extraction in this model was determined to be a solvent composition of 50% ethanol in water (*v*/*v*), with a residue-to-solvent ratio of 0.2 (*m*/*v*).

The extract used for the following analyses was obtained with the process adjusted to the parameters defined as best parameters by the experimental plan.

### 3.3. Peanut Skin Extract Composition and Bioactivity at Optimized Conditions

#### 3.3.1. Phenolic Composition of the Extract

The quantification of total phenolics using the Folin–Ciocalteu method showed that the peanut skin extract obtained under the optimal conditions contained 538 ± 14 mg GAE/g of dry extract and the extraction yield was 9.28%. The value of total phenolics in a sample can vary greatly, depending on factors such as the peanut processing method and the methodology for recovering phenolic compounds. When comparing this result with the available literature, there is a substantial improvement in the concentration of phenolic compounds. Analyzing an aqueous extract, Cordeiro-Massironi et al. [27] found 68.5 mg GAE/g of peanut skin extract. In another study, using acetone (50% *v*/*v* in H_2_O) and ethanol (90% *v*/*v* in H_2_O), Lewis et al. [28] reported 67.9 and 51.8 mg GAE/g of peanut skin, respectively. Also using an ethanolic extract, Fernandes et al. [29] found 425 mg GAE/g of peanut skin extract.

Regarding the phenolic profile, 19 phenolic compounds were identified in the hydroalcoholic extract of peanut skin obtained through UPLC-ESI-QTOF-MS/MS analysis, as can be seen in Table 6.

#### 3.3.2. Antioxidant and Antihypertensive Activity in Vitro

Table 7 shows the results of the in vitro antioxidant and antihypertensive activity of peanut skin extract. It can be observed that peanut skin extract has a high antioxidant capacity, measured through three different methods (ORAC, DPPH, and FRAP). Putra et al. [30] conducted a review covering several methods of extraction of phenolic compounds from peanut skin, evaluating their effect on antioxidant capacity. Alcohol extraction resulted in an ORAC level of 2.149 mmol TE/mg, a value lower than that found in the present study. Another study conducted by Lewis et al. [28] also reported a lower antioxidant capacity (1830 ± 58 µmol TE/ g) in a hydroethanolic (90%) peanut skin extract.

The antioxidant activity of phenolic compounds is measured as the free radical scavenging capacity and depends on the number of hydrogen atom donor sites (typically hydroxyl groups attached to aromatic rings) and the position of these active groups [31]. Polyhydric phenol groups with a high number of OH groups, commonly identified in peanut shell extracts, may have a high antioxidant power, which explains the high ORAC, DPPH, and FRAP values found in this study. In addition, it showed a high degree of inhibition of the ACE enzyme.

The Renin–Angiotensin–Aldosterone System (RAAS) reaction cascade starts with angiotensinogen release by the liver, followed by its cleavage and angiotensin I liberation by renin enzyme activity. Angiotensin I is not biologically active but, via ACE activity, it is converted to angiotensin II, which promotes the biological response by interaction with a proper receptor. This interaction activates cellular processes, such as vasoconstriction and aldosterone production, that lead to BP increasing. Aldosterone is the hormone responsible for signaling to kidneys for water and sodium reabsorption [8]. Thus, RAAS is an important mechanism for blood pressure control and is usually activated during hypotensive episodes for blood pressure increase, but its hyperactivation can lead to a hypertensive condition, which make it the main target for hypertension treatment, mainly by ACE inhibition [9]. Considering this, compounds that can inhibit this enzyme, such as some phenolic compounds, can also present antihypertensive potential.

#### 3.3.3. Effect of the Peanut Extract in ROS Generation in Caco-2 Cells

**Figure 2:**
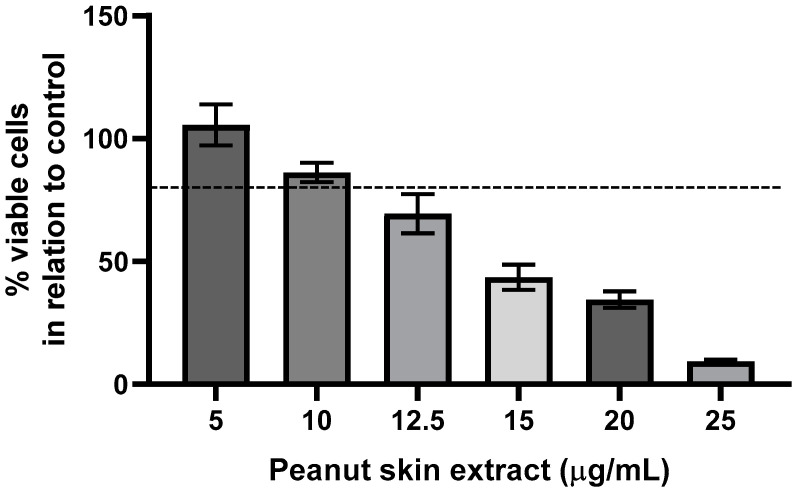
Effect of peanut skin extracts in different concentrations (5–25 µg/mL) in Caco-2 cells. Data are expressed in % of viable cells in relation to control cells as mean ± SD.

**Figure 3:**
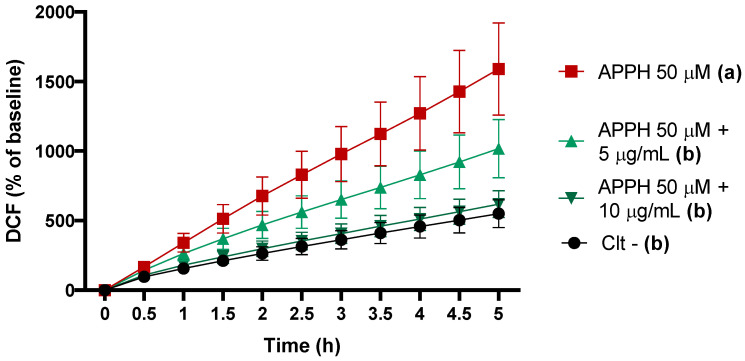
Intracellular reactive oxygen species (ROS) production in Caco-2 cells treated with peanut skin extract at 5 and 10 μg/mL in HBSS with 50 μM AAPH. Values are expressed as mean ± SD. (a) and (b) at the same time point without sharing the same letters significantly differ (*p* < 0.05), using one-way ANOVA, followed by post hoc Tukey test.

To carry out the intracellular antioxidant capacity test, the first step is always to define the safe concentrations of extract to be added to the cells. To do this, we performed the MTT assay, which determines cell viability against different sample concentrations. The cytotoxicity of the peanut skin extract was tested in Caco-2 cells for 24 h in a concentration range of 5 to 25 µg/mL. From 12.5 to 25 µg/mL, the extract reduced cell viability to less than 80%, indicating a concentration-dependent effect. Therefore, the results shown in Figure 2 indicated that it was safe to work at concentrations equal to or less than 10 µg/mL. Having defined the concentrations for the next test, we started to evaluate the modulation of ROS by the samples in intestinal epithelial cells, and the results are presented in Figure 3. The cumulative ROS in Caco-2 cells increased by 240% after 5 h of exposure to AAPH alone when compared to the control cells. When simultaneously exposed to peanut extract plus AAPH, they could reduce ROS production to similar values to those from control cells. Both doses from each extract displayed similar effects.

The dose used in the ROS assay was non-cytotoxic and exhibited strong antioxidant activity, even at low concentrations. Similar studies evaluating the cytotoxicity of peanut skin extract in various cell types—such as normal epithelial cells, rat ileum cells, monkey kidney cells, human peripheral blood mononuclear cells, and rat macrophages—reported comparable results, confirming that peanut skin extract exhibits antioxidant activity at non-cytotoxic doses [32,33]. However, it is important to highlight that the extract obtained in this study was cytotoxic in lower doses. This characteristic of the extract is likely due to its high polyphenol content. As widely reported in the literature, polyphenols are generally beneficial to health because of their potent antioxidant properties. However, in excess, they can act as pro-oxidants, potentially overwhelming cellular systems [34]. The MTT assay, used to assess cytotoxicity, is based on mitochondrial activity, which is closely linked to oxidative metabolism. We hypothesize that the cytotoxicity of peanut extract at higher doses is likely due to its elevated phenolic compound content, which may exert pro-oxidant effects on cells, overloading their mitochondria. Nonetheless, we consider this result positive, as it demonstrates that peanut extract could act as a powerful antioxidant at very low doses, supporting its potential use as an antioxidant ingredient in the food industry.

Oxidative stress is caused by the imbalance between endogenous antioxidants and oxidants (mainly ROS), favoring oxidative status. This condition is also linked to blood pressure increasing by the promotion of vascular inflammation, vasodilator oxidation, and ACE secretion increasing [35]. In that way, antioxidant capacity can also collaborate with hypertension control by reducing oxidative stress. Studies have suggested that antihypertensive potential of phenolic extracts can be attributed to their free radical scavenging capacity, as well as to endogenous antioxidant enzymes stimulation [35,36].

## 4. Conclusions

This study demonstrates an efficient method for extracting phenolic compounds from peanut skin residue using a 50% (*v*/*v*) hydroalcoholic solution. Our findings indicate that ethanol concentrations below 50% result in a significant reduction in total phenolic content. Although the decrease in total phenolic compounds was not drastic, it would still be impractical from a processing perspective. Lower ethanol concentrations would significantly increase the time and energy required for rotary evaporation, making the process more costly.

The extract obtained demonstrated strong antioxidant capacity in vitro. In addition, the peanut skin extract also showed an inhibitory capacity for the ACE enzyme, demonstrating an antihypertensive potential. To the best of our knowledge, this is the first report about peanut skin phenolic extract as a source of bioactive compounds with antihypertensive potential by this mechanism.

While the peanut skin extract exhibited cytotoxicity at higher concentrations, it effectively reduced the generation of ROS in Caco-2 intestinal cells, indicating its antioxidant potential even at low, non-cytotoxic doses. Despite the observed cytotoxicity at elevated concentrations, the extract’s efficacy at lower doses highlights its potential for safe use in antioxidant applications. This is particularly relevant as Caco-2 cells are commonly used to model the human intestinal barrier, suggesting that the extract could offer protective effects against oxidative stress in the gastrointestinal tract. The high phenolic content of the extract likely contributes to this antioxidant activity, supporting its potential as a valuable ingredient for functional foods or nutraceuticals aimed at promoting intestinal health.

## Figures and Tables

**Table 1:** Variables and level codes for DCCR varying ethanol concentration of the hydro-alcoholic extraction solution (% *v*/*v*), and the proportion of solid sample to liquid in the extraction (*m*/*v*).

Variables	Units	Levels				
		−1.41	−1	0	1	1.41
Ethanol concentration	% *v*/*v* in H_2_O	41.72	50	70	90	98.28
Waste ratio	% *m*/*v*	0.02	0.05	0.13	0.2	0.23

**Table 2:** Proximate composition of crushed peanut skin.

Component	g/100 g
Carbohydrates	48.3
Lipids	25.7
Proteins	18.6
Moisture	5.11
Ashes	2.22

**Table 3:** Analyses of variance and regression for the response of TF of the 2^2^ central composite design.

	Ethanol (% *v*/*v*)	Waste Ratio (% *m*/*v*)	TPC (µmol AGE/mg Dry Extract)		ORAC (µmol ET/g Dry Extract)	
	x_1_ (X_1_) ^A^	x_2_ (X_2_)	Experimental	Predicted ^B^	Experimental	Predicted ^B^
1	−1 (50)	−1 (0.05)	297.64	280.36	2036.70	1839.28
2	1 (90)	−1 (0.05)	318.28	278.99	1951.79	1649.5
3	−1 (50)	1 (0.2)	537.87	540.85	3163.37	3113.7
4	1 (90)	1 (0.2)	348.79	329.77	1935.48	1773.31
5	−1.41 (41.72)	0 (0.13)	429.83	432.42	2646	2751.64
6	1.41 (98.28)	0 (0.13)	248.48	282.19	1421.09	1675.04

^A^ xi is the coded value and Xi is the actual value of the ith independent variable. The conversion between xi and Xi is described in Equation (1). ^B^ Modeling predicted results for each assay.

**Table 4:** Analyses of variance and regression for the response of TF of the 2^2^ central composite design.

Source of Variation	Sum of Squares	Degrees of Freedom	Mean Square	F Test	*p*-Values
Regression	111,060	5	22,211.92	21.75	0.0021
Residual	5107	5	1021.46	-	-
Lack of fit	4426	3	1475.29	4.33	0.1933
Pure error	681	2	340.71	-	-
Total	116,167	10	-	-	-

Coefficient of determination: R2 = 0.95. a F_t 0.9; 5;5_ = 3.45.

**Table 5:** Analyses of variance and regression for the response of PRAC of the 2^2^ central composite design.

Source of Variation	Sum of Squares	Degrees of Freedom	Mean Square	F Test	*p*-Values
Regression	2,943,150	5	588,629.95	9.13	0.0149
Residual	322,222	5	64,444.40	-	-
Lack of fit	319,876	3	106,625.42	90.91	0.0109
Pure error	2346	2	1172.87	-	-
Total	3,265,372	10	-	-	-

Coefficient of determination: R^2^ = 0.90. ^a^ F_t 0.9; 5;5_ = 3.45.

**Table 6:** UPLC-ESI-QTOF-MS/MS analysis of peanut skin extract obtained under optimal conditions.

Rt (min)	Phenolic Compound	[M-H]- (*m*/*z*)	Theoretical Molecular Weight	Molecular Formula
5.672534	Protocatechuic acid	153.0164	154.02606	C_7_H_6_O_4_
7.671317	Coumaric acid isomer	163.0366	164.04679	C_9_H_8_O_3_
6.826033		163.037	164.04679	
8.439183		163.0384	164.04679	
1.134717	Sinapic acid	223.0447	224.06792	C_11_H_12_O_5_
6.672067	(+)-Catechin	289.0698	290.07848	C_15_H_14_O_6_
10.38052	(3R)-4′-Methoxy-2′,3,7-trihydroxyisoflavanone	301.0327	302.07848	C_16_H_14_O_6_
1.16575	Caffeic acid O-glucoside	341.1082	342.09453	C_15_H_18_O_9_
1.186817	Kaempferol-3-O-arabinofuranoside	417.1249	418.08944	C_20_H_18_O_10_
1.16575	Kaempferol-3-O-rhamnose isomer	431.138	432.10509	C_21_H_20_O_10_
6.991883		431.1511		
12.72048		431.1823		
13.51903	Quercetin-3-O-arabinofuranoside	433.1245	434.08436	C_20_H_18_O_11_
13.18615	(-)-Epicatechin-3-O-gallate	443.1942	442.08944	C_22_H_18_O_10_
1.186817	Apigenin 7-O-glucuronide	445.1193	446.08436	C_21_H_18_O_11_
8.04775	Isorhamnetin-3-O-glucuronide	491.0556	492.08984	C_22_H_20_O_13_
7.38705		491.0571		
13.96103	1,3-di-O-Caffeoylquinic acid	515.1268	516.1262275	C_25_H_24_O_12_
15.12023		515.1428		
12.94457		515.1769		
1.0889	Apigenin-6-C-glucoside-8-C-arabinoside	563.1819	564.14735	C_26_H_28_O_14_
7.403283	Kaempferol-7-O-neohesperidoside	593.1272	594.15792	C_27_H_30_O_15_
9.628616		593.1443		
11.5051	Isorhamnetin 3-O-rutinoside	623.0273	624.16848	C_28_H_32_O_16_
9.73365		623.1608		
8.079917	Kaempferol-3-sophoroside-7-glucoside	771.1964	772.20565	C_33_H_40_O_21_
7.148567	Epicatechin-(4beta->8)-epicatechin-(4beta->8)-catechin	867.1927		C_45_H_89_O_18_
6.3614	Kaempferol-3-sophorotrioside-7-glucoside	933.1758	934.25848	C_39_H_50_O_26_

**Table 7:** Antioxidant capacity in vitro of extract obtained from peanut skin.

Sample	ORAC (μmol TE/g d.e.)	DPPH (μmol TE/g of d.e.)	FRAP (μmol TE/g of d.e.)	ACE Inhibition (%)
Peanut skin extract	3163 ± 308	1539 ± 8	2196 ± 94	95.4 ± 0.1

Results are expressed as mean ± SD. TE: Trolox Equivalent. d.e.: dry extract.

## Data Availability

The original contributions presented in the study are included in the article, further inquiries can be directed to the corresponding author.
